# The effect of a customised digital adherence tool on HIV treatment outcomes in young people living with HIV (YPLHIV) in Blantyre, Malawi: a protocol for a randomised controlled trial

**DOI:** 10.1186/s13063-023-07496-6

**Published:** 2023-08-15

**Authors:** Takondwa Charles Msosa, Iraseni Swai, Marion Sumari-de Boer, Kennedy Ngowi, Tobias F. Rinke de Wit, Rob Aarnoutse, Marriott Nliwasa

**Affiliations:** 1 Helse Nord Tuberculosis Initiative, Department of Pathology, Kamuzu University of Health Sciences, Blantyre, Malawi; 2 Department of Global Health, Amsterdam UMC, Location University of Amsterdam, Amsterdam Institute for Global Health and Development, Meibergdreef 9, Amsterdam, the Netherlands; 3 Kilimanjaro Clinical Research Institute, Moshi, Tanzania; 4 Institute of Public Health, Kilimanjaro Christian Medical University College, Moshi, Tanzania; 5 Knowledge, Innovation and Technology Group, Wageningen University & Research, Droevendaalsesteeg, the Netherlands; 6 Amsterdam Institute of Global Health and Development, Amsterdam, the Netherlands; 7 PharmAccess Foundation, Amsterdam, the Netherlands; 8 Department of Pharmacy, Radboud University Medical Center, Research Institute for Medical Innovation, Nijmegen, the Netherlands

**Keywords:** Digital health, HIV, Young people living with HIV, ART adherence, Viral load

## Abstract

**Background:**

People living with HIV (PLHIV) have to take lifelong antiretroviral treatment, which is often challenging. Young people living with HIV (YPLHIV) have the lowest viral load suppression rates in Malawi and globally, mostly due to poor treatment adherence. This is a result of complex interactions of multiple factors unique to this demographic group. The use of digital health interventions, such as real-time medication monitor (RTMM)-based digital adherence tools (DATs), could improve ART adherence in YPLHIV and subsequently improve viral load suppression which in turn could lead to reduced HIV-associated morbidity and mortality.

**Aim:**

To provide the evidence base for a digital adherence intervention to improve treatment outcomes in YPLHIV on ART.

**Objectives:**

1. The primary objective is to determine the efficacy of a customised DAT compared to the standard of care in improving ART adherence in YPLHIV.

2. The secondary objective is to determine the efficacy of the customised DAT compared to the standard of care in improving viral load suppression in YPLHIV.

**Methodology:**

This will be a parallel open-label randomised control controlled two-arm trial in which non-adherent YPLHIV in selected ART facilities in Blantyre will be randomised in a 1:1 ratio to a customised DAT and standard care arms and followed up for 9 months. The primary outcome is the proportion adherent at 9 months (> = 95% by pill count), and the secondary outcome is the proportion with viral load suppressed at 9 months (< 200 copies/ml).

**Discussion:**

There is a paucity of good quality evidence on effective digital health interventions to improve ART adherence and viral load suppression in YPLHIV globally and particularly in HIV high-burden settings like Malawi. This study will provide good-quality evidence on the effectiveness of a customised DAT in improving ART adherence and viral load suppression in this important demographic.

**Trial registration:**

The trial has been registered in the Pan African Clinical Trials Registry number: PACTR202303867267716 on 23 March 2023 and can be accessed through the following URL: https://pactr.samrc.ac.za/TrialDisplay.aspx?TrialID=25424. All items from the WHO Trial Registration Data Set are described in this manuscript.

**Supplementary Information:**

The online version contains supplementary material available at 10.1186/s13063-023-07496-6.

## Background

As of 2021, approximately 38.4 million people were living with HIV/AIDS (PLHIV) worldwide, of whom 28.7 million had access to antiretroviral therapy (ART) [1]. According to global estimates of 2021, about 3,300,000 young people (15–24 years of age) were living with HIV (YPLHIV), representing 8.6% of global HIV positives [1].

In Malawi, as of 2021, it was estimated that there were approximately 990,000 PLHIV, representing a population prevalence of 8.9% [1]. Furthermore, there were approximately 100,000 YPLHIV, a proportion of about 10% of PLHIV in Malawi [1]. Additionally, HIV prevalence in young people was estimated to be 2.5%, much higher than the global prevalence of 0.3% [1]. Going further, HIV incidence in young people was estimated to be 1.69 per 1000, much higher than the global estimate of 0.34 per 1000 [1]. In terms of HIV-related deaths in Malawi, as of 2021, it was estimated that YPLHIV contributed to approximately 1400 deaths out of a total of 7900 deaths, further emphasising the need to improve prevention and treatment interventions in this demographic [1].

The scale-up of ART has led to improved treatment outcomes in PLHIV. Early case detection, rapid ART initiation, and support of treatment adherence are the most important interventions reducing associated morbidity and mortality [2]. However, multiple studies have demonstrated significant gaps across the HIV treatment cascade in YPLHIV, resulting in less-than-ideal outcomes regarding linkage and retention in care and viral load suppression [2].

Viral load suppression is consistently lower in YPLHIV compared to other age groups. A cross-sectional study conducted in 2016 in southern Malawi on virological non-suppression in YPLHIV estimated that approximately 39% of YPLHIV were virally non-suppressed [3]. Furthermore, multiple Population-Based HIV Impact Assessments (PHIAs) in sub-Saharan Africa have backed this trend. For example, results from the Malawi Population-Based HIV Impact Assessment of 2020 showed that YPLHIV had a much lower viral load suppression prevalence as compared to other age groups; males had a viral load suppression prevalence of 75%, and females had a viral load suppression prevalence of 73.2% as compared to the national average of 88.3% [4].

Suboptimal adherence to an ART regimen is associated with a greater risk of developing resistance to antiretroviral agents and increases the risk of HIV-related morbidity and mortality and progression to AIDS [5]. Therefore, recognising the importance of ART adherence, UNAIDS recommends an adherence target of >  = 90% to combat HIV infections globally, while other studies recommend an adherence of 95% to achieve viral load suppression [5, 6]. Despite such recommended high adherence rates, several other studies have shown that a medication adherence rate of >  = 80% is sufficient to achieve viral load suppression [5, 7, 8]. The differences between these studies may relate to differences between antiretroviral drugs in so-called forgiveness for non-adherence. In any case, the importance of high ART adherence cannot be overstated in reducing HIV-associated morbidity, mortality, drug resistance, and transmission.

There is significant evidence that YPLHIV are the least adherent to ART as compared to other age groups, probably due to their formative stage of psychosocial and cognitive development in which they experience significant biological, psychological, and psychosocial transitions, rendered more difficult by the addition of an HIV diagnosis [3, 9]. Subsequently, sub-optimal ART adherence in YPLHIV leads to poor virological suppression rates and treatment outcomes.

Various digital health interventions have been implemented to improve ART adherence and viral load suppression in PLHIV, and some have proven to be effective [10]. Digital health technologies like real-time medication monitors (RTMM, i.e. pill box that records opening of the box) used in conjunction with SMS reminders and customised adherence feedback are promising tools for improving ART adherence and viral load suppression in YPLHIV. For example, a systematic review of digital health interventions in YPLHIV demonstrated that phone-based interventions might improve ART adherence and viral load suppression in adolescents [11].

Therefore, in this article, we describe the methodology of a randomised controlled trial to evaluate the effectiveness of a customised digital adherence tool (DAT) on ART adherence and viral load suppression in YPLHIV on ART in Blantyre, Malawi. We hypothesise that the RTMMs plus reminder text messages and tailored adherence feedback (RTMM-based DAT) could improve ART adherence and viral load suppression in YPLHIV on ART when compared to the standard of care which is intensive adherence counselling.

## Methods

The trial protocol has been written according to the SPIRIT reporting guidelines [12].

### Aim and objectives

#### Aim

The aim is to provide the evidence base for a digital adherence intervention that could improve treatment response and outcomes in YPLHIV on ART.

#### Objectives

1. The primary objective is to determine the efficacy of the customised DAT compared to the standard of care in improving ART adherence in YPLHIV.
2. The secondary objective is to determine the efficacy of the customised DAT compared to the standard of care in improving viral load suppression in YPLHIV.

### Trial design

**Fig. 1:**
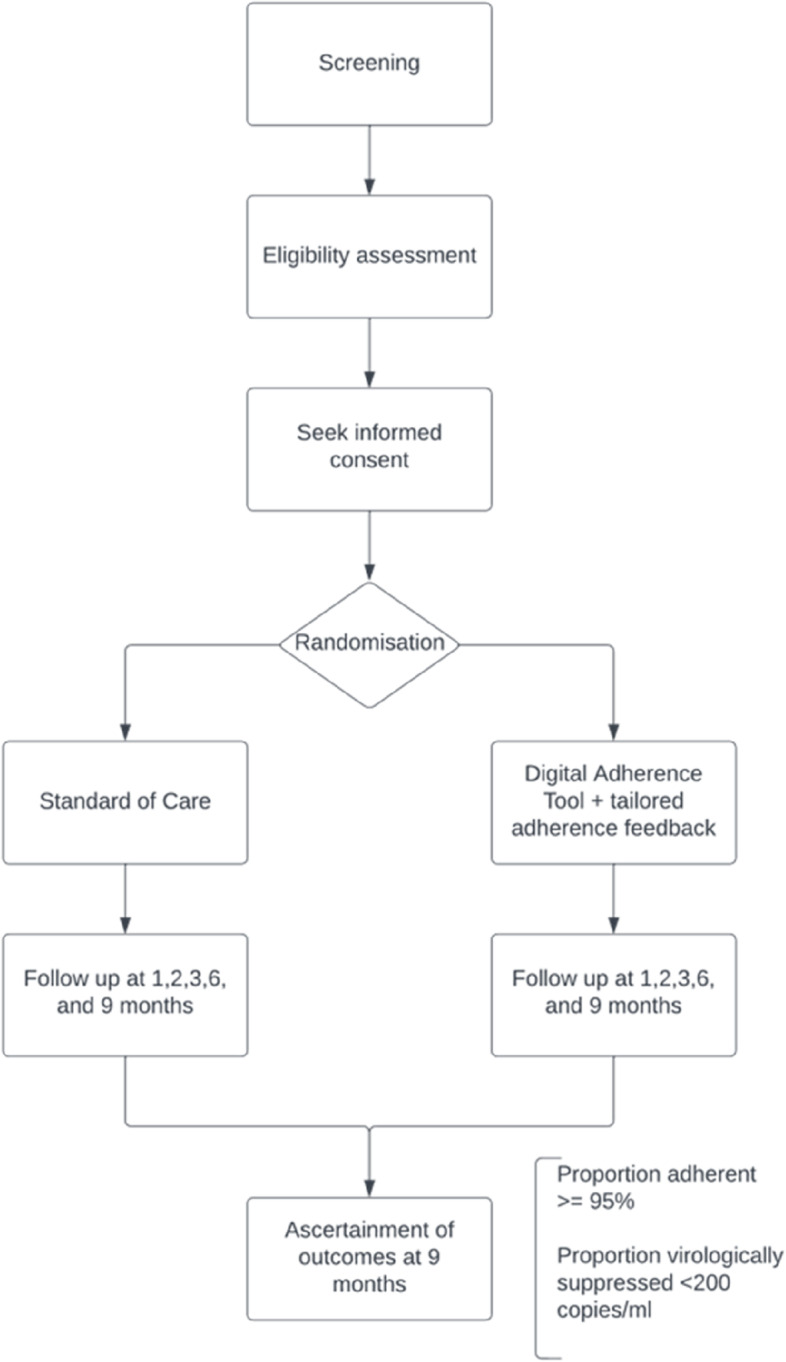
Flowchart of events

This will be a parallel open-label randomised controlled 2-arm trial in which YPLHIV will be randomised in a 1:1 ratio to the DAT and standard care (SOC) arms and followed up for 9 months. This design will enable us to evaluate the superiority of the intervention compared to the SOC. Figure 1 shows the flowchart of events.

### Study setting

The study will occur in Blantyre, Malawi, in the ART clinics of the following primary health facilities: Bangwe, Ndirande, Limbe, Chilomoni, and South Lunzu. These facilities were chosen because they have the highest number of YPLHIV in Blantyre district.

### Eligibility criteria

The study population will be people living with HIV aged between 15 and 24 years (YPLHIV) on ART registered at the study sites. Participants will be YPLHIV who are non-adherent to ART, i.e. less than 80% of prescribed pill intake based on pharmacy refill counts.

To be included in the study, participants must be on first-line ART regimen (tenofovir/lamuvidine/dolutegravir) or (tenofovir/lamuvidine/efavirenz) according to local guidelines, must have had full disclosure of their HIV status, must be able to read and understand Chichewa or English, and must be competent in the use of mobile devices.

Exclusion criteria include hospitalisation at the time of study entry, prior participation in digital adherence health research, enrolment in other HIV-related studies, and treatment for a comorbidity that significantly increases the participants’ pill burden.

### Interventions

#### Standard of care

The standard of care involves routine ART clinic procedures and protocols as stipulated by the national guidelines [13]. These include intensive adherence counselling and targeted viral load testing after 3 months of optimal adherence for these clinic attendees for ART clinic attendees who are non-adherent and/or virologically non-suppressed or at risk of virological failure.

#### RTMM-based digital adherence tool

The RTMM is a pillbox developed by Wisepill™ containing a SIM card that sends data to a central server through the worldwide general packet radio service (GPRS) network. The pillbox can hold approximately 30 large pills or 60 small pills [14]. In the context of this study, the participants will store their ARV fixed combination tablets in the pillbox.

Data about openings will be immediately sent to and stored on a secure server. The data will include information about the opening time, the dispenser’s identification number, and battery and signal strength specifications. If medication is not taken within one hour of the prescribed time, the participant will receive a text message reminder. The messages will be neutral and unrelated to the HIV status of the recipients.

Participants in the intervention arm will also receive customised adherence feedback based on server-stored adherence data at every scheduled clinic visit. This feedback will be provided by a psychosocial counsellor or an ART nurse or clinician with the help of a research assistant. The feedback sessions will take into consideration the complex interaction of multiple factors unique to the demographic group of YLHIV. During these discussions, participants will go through the stages of precontemplation, contemplation, preparation, action, and evaluation according to the stages of change model. This approach has been adopted from a similar trial conducted by Sumari-de Boer et al. in Tanzania [15]. Participants will be asked about their opinion regarding their self-reported and pharmacy refill adherence since the previous visit (precontemplation), followed by being shown their adherence report from the RTMM server on which participants will be asked to reflect (contemplation). A discussion on possible barriers for adherence and next steps will then be undertaken and a target will be set for the next visit (preparation). After the feedback, participants will be expected to have increased intention to adhere, which should be followed by improved adherence (action). During the next visit, the same process will be repeated, including the evaluation of the preceding period. Lastly, since the intervention is an adherence aid, participants will undergo medical care as prescribed by ART clinic staff and according to Malawi HIV treatment guidelines. Therefore, participants enrolled in this study will not be barred from receiving any concomitant care.

### Outcomes

The primary outcome is the proportion adherent at 9 months, which is the percentage of prescribed drugs consumed as ascertained on the clinic visit. These will be determined at 1, 2,3, 6, and 9 months. Both self-reported adherence and pharmacy refill counts will be measured at each scheduled visit; these methods have been described in a similar trial by Sumari de Boer et al. [15]. A cut-off of >  = 95% of prescribed pill intake based on the Malawi National Guidelines will be used to classify participants as to whether they were adherent or non-adherent [13].

The secondary outcome is the proportion virologically suppressed at 9 months. A blood plasma sample will be collected for all study participants at baseline and at nine months for viral load testing using real-time polymerase chain reaction (rtPCR). Viral load suppression will be defined by a cut-off of < 200 copies/ml as this cut-off has been shown to have a zero transmission risk and is of public health importance in terms of epidemic control [16, 17].

Serum samples for viral load will be collected by the research assistant or clinic staff. We will follow standard care procedures in obtaining blood samples. These samples will then be sent to the Kamuzu University of Health Sciences Microbiology Laboratory for processing within 24 h.

### Participant timeline

Study procedures and timelines are also displayed in Table 1.

**Table 1:**
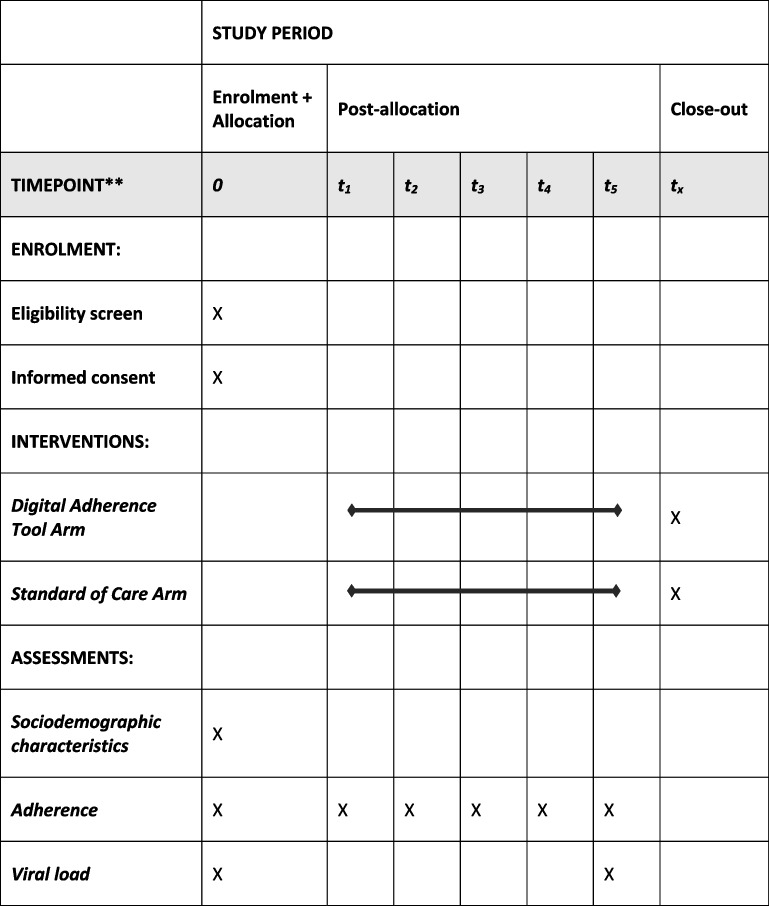
SPIRIT figure for study procedures and events

** t_*1*_ = 1 month, *t*_*2*_ = 2 months, *t*_*3*_ = 3 months, *t*_*4*_ = 6 months, *t*_*5*_ = 9 months, *t*_*x*_ = 16 months

### Sample size

The sample size calculation is an estimation based on ART adherence. At endline, participants will be classified into 2 categories: adherent, and non-adherent based on pill count. The study intends to demonstrate a difference of 15% in adherent participants between the intervention arm (90%) and the control arm (75%). Since there is no recent published literature on baseline adherence levels in YPLHIV in Malawi, virological suppression data from the recent Malawi Population-based HIV Impact Assessment 2020–2021 [13] and an observational study on adherence and virological suppression conducted in the southern districts of Malawi [3] have been used as a proxy for ART adherence in this demographic, hence the 75% expected adherence proportion at endline for the SOC arm. With a power of 80% and *α* = 0.05, we will need 100 participants in each arm. Considering 20% loss-to-follow-up, the sample size needed is 120 participants in each arm. The R statistical package “pwr” version 1.3 was used to compute the sample size [18].

### Recruitment

Since participants will be recruited from ART clinics, research assistants in addition to, ART clinic personnel and research assistants will be trained on the protocol and will be responsible for identifying eligible clients. In addition, posters and flyers will be displayed in ART clinics to raise awareness of the study.

### Allocation to study arms

For allocation to study arms, stratified block randomisation with variable block sizes will be employed. Allocation cards will be printed and stored in envelopes that will be kept in secure lockable cabinets at the recruitment sites. During the screening/first visit, the research assistant will assign and enrol study participants. The participants’ gender and age will be stratifying factors. The data manager will be responsible for creating allocation cards, while the research assistants will assign participants to each study arm.

Furthermore, it will not be feasible to blind participants and site investigators to arm allocation due to the nature of the intervention. However, for the assessment of the secondary endpoint, laboratory staff processing viral loads will be blinded to arm allocation. Furthermore, the trial statistician will be blinded to study arms when assessing the primary and secondary outcomes.

### Data collection methods

A research assistant will screen for eligibility, solicit consent, and enrol participants. The research assistant will hand an informed consent form to a potential participant in their preferred language and collect baseline characteristics and information on outcomes when applicable.

To minimise loss to follow-up, at enrolment, a geolocation of participants’ place of residence using the ePAL android app, a high-resolution mapping system validated in Blantyre will be used should participants consent. We will also record up to 3 contact phone numbers of the participant and their nominated friends and relatives. Should a participant miss a study visit, we will contact them by phone or by visiting them at home to encourage them to attend the study visit before the expiry of the prescribed visit window.

### Data management

Standardised, pre-tested questionnaires will collect data from participants at every visit. The electronic data collection tools will be designed using REDCap. All paper records will be kept in a locked space and will only be accessible to approved study staff. Furthermore, for the real-time medication monitoring, anonymised data will be stored on a secure server.

Routine data quality assurance processes will be conducted from time to time. Queries will be raised in the database to resolve data recording inconsistencies. In addition, a random sample of individual participant records in the database will be cross-checked with source documents for the correctness of data entry procedures during quality assurance monitoring.

### Statistical analysis

All analyses will be done using R [19]. Reporting will follow the CONSORT guidelines [20]. A flowchart will describe the inclusion and follow-up of participants by study arm. Baseline characteristics and outcomes will be described by the study arm with summary statistics for continuous variables and number and percentage for categorical variables.

The primary and secondary analyses for this study will be the comparison of adherence and viral load suppression proportions between study arms at month 9. A 2-proportion *z*-test will be utilised to test for statistical significance and 95% confidence intervals and *p*-values will be computed. The following analysis sets will be used for both the primary and secondary outcomes:

- Modified intention-to-treat (mITT): anyone found to be ineligible after randomisation will be excluded
- Per-protocol (PP) set: this set includes all participants who completed the study without a major protocol deviation

Furthermore, additional analyses of both the primary and secondary outcome will involve the use a logistic regression model adjusted for the pre-specified randomisation stratification factors (sex and age) [21] and important baseline characteristics if found to be unbalanced between study arms [22]. Furthermore, to determine the efficacy of the intervention over time, a multilevel logistic regression model will be utilised. A detailed statistical analysis plan will be submitted later. Lastly, missingness will be defined for important variables, and if appropriate, multiple imputation will be employed. Lastly, no interim analysis is planned, and subgroup analyses will be described in a detailed statistical plan.

### Study management and monitoring

A trial management group (TMG) will also be appointed and will be responsible for overseeing the progress of the trial. Furthermore, a data safety management board (DSMB) will be set up before commencing the trial. The DSMB will provide an independent review of the study’s conduct, progress, and findings. The study will be monitored just before commencing enrolment, then periodically by a monitoring team from the Kamuzu University of Health Sciences.

### Management and reporting of adverse events

Adverse events will be classified according to the International Council for Harmonisation of Technical Requirements for Pharmaceuticals for Human Use (ICH)—Good Clinical Practice (GCP) classifications. An event will be considered as serious if it results in death, is life-threatening, results in hospitalisation, or results in domestic violence. The causal relationship of the event to the intervention will be assessed and classified according to standard definitions as “not related”, “possibly related”, “probably related”, and “definitely related.”

Due to the nature of the intervention, we do not expect serious adverse events (SAEs); however, a log of events will be developed including standard operating procedures (SOPs) on how to resolve them in accordance with national guidelines. Adverse events will be solicited at each study visit, and participants will be instructed to contact the trial coordinator should they experience any events of concern. Furthermore, the intervention will be ceased upon the participant’s request or in case the investigator notices that the participant is incapable of using the intervention or in the event of a SAE.

### Protocol amendments and deviations

Protocol amendments will first be sent to the trial sponsors for permission. Thereafter, we will seek ethical approval from the local ethical board, the College of Medicine Research and Ethics Committee (COMREC). Only after approval by COMREC will the amended protocol be implemented. All protocol violations and deviations will be reported and resolved according to standard operating procedures (SOPs) that will be developed and also according to funder and ethics board regulations.

### Dissemination

The results of this trial will be disseminated at relevant local and international conferences. Additionally, the results of this study will be submitted for publication in open-access medical journals and be shared with relevant local and international stakeholders. Lastly, information leaflets describing the results of this study will be developed and disseminated to study participants and participating clinics.

## Discussion

Several studies have been conducted on the effectiveness of RTMM-based DATs in improving ART adherence and viral load suppression in PLHIV. For example, in China, a randomised controlled trial on ART initiates showed that a combination of RTMMs and reminder text messages significantly improved ART adherence but not virological suppression [23]. However, a randomised controlled trial in South Africa on the effectiveness of RTMMs in improving ART adherence in ART naïve participants showed no difference in adherence and virological suppression between the RTMM arm and the standard of care (SOC) [24]. Similar findings were observed in another trial in Tanzania that enrolled adults on ART for at least 6 months [15]. Contrasting other studies that showed no effect, a per-protocol analysis of a randomised control trial on the effectiveness of RTMM in improving ART adherence in pregnant and postpartum women living with HIV in Uganda showed a significant improvement in ART adherence in the intervention arm as compared to the standard of care [25].

Therefore, even though the evidence is not definitive on employing RTMM-based DATs in PLHIV, this intervention could prove to be effective in improving ART adherence and virological suppression in YPLHIV. Furthermore, to the best of our knowledge, this will be one of the first trials to investigate the effectiveness of this DAT in YPLHIV and will provide high-quality evidence in a group that is often neglected in adherence research [11].

The study design has several strengths. First, the trial will be conducted in primary health facilities within Blantyre and with minimal deviation from normal clinic procedures, which will provide important information on probable implementation effectiveness, barriers, and facilitators. Furthermore, in addition to adherence by pill counting as a primary outcome, the addition of viral load suppression as a secondary outcome will offer high-quality evidence on the effectiveness of the intervention on an important indicator.

However, there are several limitations. First, this study will only recruit participants that can read and write; therefore, the external validity of the study will be negatively impacted as many ART clinics have a significant number of clients that have low literacy. Furthermore, medication adherence will be measured by pharmacy refill counts and self-reported adherence which are prone to patient manipulation and observer or recall bias which might lead to non-differential misclassification of the outcome.

Going further, concerning the secondary outcome, viral load cannot be used as an instrumental variable to directly measure adherence due to the heterogeneity and non-linearity of the relationship between ART adherence and viral load and the also the effect of HIV drug resistance on viral loads [26, 27]. Lastly, the nature of the intervention will not allow for blinding which might lead to a change in behavioural patterns of the participants and clinic staff because of the intervention which might not be observed in its real-world implementation.

In conclusion, we believe that the results of this study will be crucial in identifying and developing effective adherence interventions for YPLHIV. Moreover, we will be able to provide novel high-quality evidence on the effectiveness of the RTMM-based DAT in Malawi and similar settings, therefore providing essential information on effective interventions for improving HIV treatment outcomes in YPLHIV.

### Supplementary Information

**Additional file 1.** Reporting checklist for protocol of a clinical trial.

## Data Availability

Data will be made available according to applicable Malawian law and guidelines and a data sharing agreement form will have to be solicited.

## Abbreviations

ART: Antiretroviral therapy
COMREC: College of Medicine Research and Ethics Committee
DAT: Digital adherence tool
DSMB: Data safety management board
HIV: Human immune virus
PLHIV: People living with HIV
RTMM: Real-time medication monitor
SOC: Standard of care
SOPs: Standard operating procedures
TMG: Trial Management Group
YPLHIV: Young people living with HIV
